# Robot-Assisted Stair Climbing Training on Postural Control and Sensory Integration Processes in Chronic Post-stroke Patients: A Randomized Controlled Clinical Trial

**DOI:** 10.3389/fnins.2019.01143

**Published:** 2019-10-24

**Authors:** Marialuisa Gandolfi, Nicola Valè, Eleonora Dimitrova, Maria Elisabetta Zanolin, Nicola Mattiuz, Elisa Battistuzzi, Marcello Beccari, Christian Geroin, Alessandro Picelli, Andreas Waldner, Nicola Smania

**Affiliations:** ^1^Department of Neurosciences, Biomedicine and Movement Sciences, University of Verona, Verona, Italy; ^2^UOC Neurorehabilitation, AOUI Verona, Verona, Italy; ^3^Unit of Epidemiology and Medical Statistics, University of Verona, Verona, Italy; ^4^Department of Neurological Rehabilitation, Private Hospital Villa Melitta, Bolzano, Italy

**Keywords:** sensory feedback, proprioception, postural balance, motor skill disorders, sensory function

## Abstract

**Background:**

Postural control disturbances are one of the important causes of disability in stroke patients affecting balance and mobility. The impairment of sensory input integration from visual, somatosensory and vestibular systems contributes to postural control disorders in post-stroke patients. Robot-assisted gait training may be considered a valuable tool in improving gait and postural control abnormalities.

**Objective:**

The primary aim of the study was to compare the effects of robot-assisted stair climbing training against sensory integration balance training on static and dynamic balance in chronic stroke patients. The secondary aims were to compare the training effects on sensory integration processes and mobility.

**Methods:**

This single-blind, randomized, controlled trial involved 32 chronic stroke outpatients with postural instability. The experimental group (EG, *n* = 16) received robot-assisted stair climbing training. The control group (*n* = 16) received sensory integration balance training. Training protocols lasted for 5 weeks (50 min/session, two sessions/week). Before, after, and at 1-month follow-up, a blinded rater evaluated patients using a comprehensive test battery. Primary outcome: Berg Balance Scale (BBS). Secondary outcomes:10-meter walking test, 6-min walking test, Dynamic gait index (DGI), stair climbing test (SCT) up and down, the Time Up and Go, and length of sway and sway area of the Center of Pressure (CoP) assessed using the stabilometric assessment.

**Results:**

There was a non-significant main effect of group on primary and secondary outcomes. A significant Time × Group interaction was measured on 6-min walking test (*p* = 0.013) and on posturographic outcomes (*p* = 0.005). *Post hoc* within-group analysis showed only in the EG a significant reduction of sway area and the CoP length on compliant surface in the eyes-closed and dome conditions.

**Conclusion:**

Postural control disorders in patients with chronic stroke may be ameliorated by robot-assisted stair climbing training and sensory integration balance training. The robot-assisted stair climbing training contributed to improving sensorimotor integration processes on compliant surfaces. Clinical trial registration (NCT03566901).

## Introduction

Postural control disturbances are one of the leading causes of disability in stroke patients, leading to problems with transferring, maintaining body position, mobility, and walking (Bruni et al., 2018). Therefore, the recovery of postural control is one of the main goals of post-stroke patients. Various and mixed components (i.e., weakness, joint limitation, alteration of tone, loss of movement coordination and sensory organization components) can affect postural control. Indeed, the challenge is to determine the relative weight placed on each of these factors and their interaction to plan specific rehabilitation programs (Bonan et al., 2004).

The two functional goals of postural control are postural orientation and equilibrium. The former involves the active alignment of the trunk and head to gravity, the base of support, visual surround and an internal reference. The latter involves the coordination of movement strategies to stabilize the center of body mass during self-initiated and externally triggered stability perturbations. Postural control during static and dynamic conditions requires a complex interaction between musculoskeletal and neural systems (Horak, 2006). Musculoskeletal components include biomechanical constraints such as the joint range of motion, muscle properties and limits of stability (Horak, 2006). Neural components include sensory and perceptual processes, motor processes involved in organizing muscles into neuromuscular synergies, and higher-level processes essential to plan and execute actions requiring postural control (Shumway-Cook and Woollacott, 2012). A disorder in any of these systems may affect postural control during static (in quite stance) and dynamic (gait) tasks and increase the risk of falling (Horak, 2006).

Literature emphasized the role of impairments of sensory input integration from visual, somatosensory and vestibular systems in leading to postural control disorders in post-stroke patients (Bonan et al., 2004; Smania et al., 2008). Healthy persons rely on somatosensory (70%), vision (10%) and vestibular (20%) information when standing on a firm base of support in a well-lit environment (Peterka, 2002). Conversely, in quite stance on an unstable surface, they increase sensory weighting to vestibular and vision information as they decrease their dependence on surface somatosensory inputs for postural orientation (Peterka, 2002). Bonan et al. (2004) investigate whether post-stroke postural control disturbances may be caused by the inability to select the pertinent somatosensory, vestibular or visual information. Forty patients with hemiplegia after a single hemisphere chronic stroke (at least 12 months) performed computerized dynamic posturography to assess the patient’s ability to use sensory inputs separately and to suppress inaccurate inputs in case of sensory conflict. Six sensory conditions were assessed by an equilibrium score, as a measure of body stability. Results show that patients with hemiplegia seem to rely mostly on visual input. In conditions of altered somatosensory information, with visual deprivation or visuo-vestibular conflict, the patient’s performance was significantly lower than healthy subjects. The mechanism of this excessive visual reliance remains unclear. However, higher-level inability to select the appropriate sensory input rather than to elementary sensory impairment has been advocated as a potential mechanism of action (Bonan et al., 2004).

Sensory strategies and sensory reweighting processes are essential to generate effective movement strategies (ankle, hip, and stepping strategies) which can be resolved through feed-back or feed-forward postural adjustments. The cerebral cortex shapes these postural responses both directly via corticospinal loops and indirectly via the brainstem centers (Jacobs and Horak, 2007). Moreover, the cerebellar- and basal ganglia-cortical loop is responsible for adapting postural responses according to prior experience and for optimizing postural responses, respectively (Jacobs and Horak, 2007).

Rehabilitation is the cornerstone in the management of postural control disorders in post-stroke patients (Pollock et al., 2014). To date, no one physical rehabilitation approach can be considered more effective than any other approach (Pollock et al., 2014). Specific treatments should be chosen according to the individual requirements and the evidence available for that specific treatment. Moreover, it appears to be most beneficial a mixture of different treatment for an individual patient (Pollock et al., 2014). Considering that, rehabilitation involving repetitive, high intensity, task-specific exercises is the pathway for restoring motor function after stroke (Mehrholz et al., 2013; Lo et al., 2017) robotic assistive devices for gait training have been progressively being used in neurorehabilitation to Sung et al. (2017). In the current literature, three primary evidence have been reported.

Firstly, a recent literature review highlights that robot-assisted gait training is advantageous as add-on therapy in stroke rehabilitation, as it adds special therapeutic effects that could not be afforded by conventional therapy alone (Morone et al., 2017; Sung et al., 2017). Specifically, robot-assisted gait training was beneficial for improving motor recovery, gait function, and postural control in post-stroke patients (Morone et al., 2017; Sung et al., 2017). Stroke patients who received physiotherapy treatment in combination with robotic devices were more likely to reach better outcomes compared to patients who received conventional training alone (Bruni et al., 2018).

Second, the systematic review by Swinnen et al. (2014) supported the use of robot-assisted gait therapy to improve postural control in subacute and chronic stroke patients. A wide variability among studies was reported about the robotic-device system and the therapy doses (3–5 times per week, 3–10 weeks, 12–25 sessions). However, significant improvements (Cohen’s *d* = 0.01 to 3.01) in postural control scores measured with the Berg Balance Scale (BBS), the Tinetti test, postural sway tests, and the Timed Up and Go (TUG) test were found after robot-assisted gait training. Interestingly, in five studies an end-effector device (gait trainer) was used (Peurala et al., 2005; Tong et al., 2006; Dias et al., 2007; Ng et al., 2008; Conesa et al., 2012). In two study, the exoskeleton was used (Hidler et al., 2009; Westlake and Patten, 2009). In one study, a single joint wearable knee orthosis was used (Wong et al., 2012). Because the limited number of studies available and methodological differences among them, more specific randomized controlled trial in specific populations are necessary to draw stronger conclusions (Swinnen et al., 2014).

Finally, technological and scientific development has led to the implementation of robotic devices specifically designed to overcome the motor limitation in different tasks. With this perspective, the robot-assisted end-effector-based stair climbing (RASC) is a promising approach to facilitate task-specific activity and cardiovascular stress (Hesse et al., 2010, 2012; Tomelleri et al., 2011; Stoller et al., 2014, 2016; Mazzoleni et al., 2017).

To date, no studies have been performed on the effects of RASC training in improving postural control and sensory integration processes in chronic post-stroke patients.

The primary aim of the study was to compare the effects of robot-assisted stair climbing training against sensory integration balance training on static and dynamic balance in chronic stroke patients. The secondary aims were to compare the training effects on sensory integration processes and mobility. The hypothesis was that the task-specific and repetitive robot-assisted stairs climbing training might act as sensory integration balance training, improving postural control because sensorimotor integration processes are essential for balance and walking.

## Materials and Methods

### Trial Design

A single-blind randomized clinical trial (robot-assisted stair climbing training – RASCT) and control group (sensory integration balance training – SIBT). The study was conducted based on the Declaration of Helsinki. The guidelines for Good Clinical Practice, and the Consolidated Standards of Reporting Trials (CONSORT), were followed. The local Ethics Committee “Nucleo ricerca clinica–Research and Biostatistic Support Unit” (1442CESC) approved the study, which was registered at clinical trial (NCT03566901).

### Participants

Consecutive chronic post-stroke outpatients referring to the Neurorehabilitation Unit (AOUI Verona) were assessed for eligibility from October 2017 to November 2018. Inclusion criteria were: age ≥ 18 years, first-ever ischemic or hemorrhagic stroke as documented by a magnetic resonance imaging or a computerized tomography scan; more than or equal to 6 months post stroke; ability to stand for at least 1 min without arm support; positive Pull test; Mini-Mental State Evaluation (MMSE) score ≥ 24/30; ability to walk independently for at least 10 m without walking aids; VAS score < 7/10 at lower limbs. Exclusion criteria were: severe visual, cognitive or cardiovascular dysfunction; deep venous thrombosis; lower limb spasticity <2 on the Modified Ashworth Scale; Botulinum toxin injection in the lower limb in the 3 months before the enrollment and during the study; other concomitant neurological or orthopedic diseases interfering with balance and ambulation. Patients gave their written, informed consent after being informed about the experimental nature of the study. They were not allowed to undergo any rehabilitation intervention during the month before the recruitment.

### Interventions

Patients underwent 10 individual rehabilitation sessions as outpatients (2 days/week, 5 weeks) at the Neurorehabilitation Unit (AOUI Verona). Each rehabilitation sessions lasted 50 min.

### Experimental Group

**FIGURE 1:**
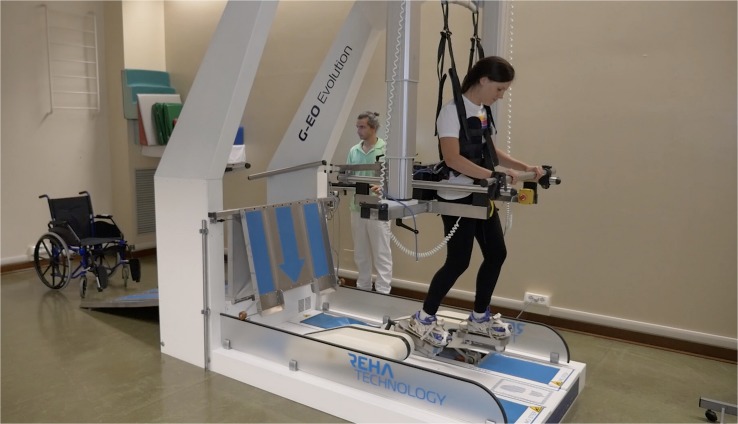
The G-EO system used in the Robot-Assisted Stair-Climbing Training (Written informed consent was obtained from the individual pictured, for the publication of this image).

Patients underwent Robot-Assisted Stair-Climbing Training (RASCT) with the G-EO system device (Reha Technology, Olten, Switzerland) (Figure 1). This end-effector robotic device can reproduce the gait pattern and realistically simulates the ability to carry out stairs up and down. The patients stood with feet secured to two foot-plates whose kinetics and kinematics parameters of the movement were adjustable. The foot-plates have three degrees of freedom each, allowing to control the step length and height and the foot-plates angles. The maximum step length corresponds to 550 mm, and the maximum achievable step height is 400 mm. The maximum angle of rotation is ± 90°. This angle controlled the plantar- and dorsiflexion of the ankles during the steps. The maximum foot-plates speed is 2.3 km/h. A physiotherapist set the pace and step length according to the patient’s impairment and the improvements achieved. Patients were secured by a harness fixed to an electric patient lift system. This system helped patients to be sustained during the walking or stair climbing task. Moreover, the G-EO system provides real-time feedback on the patient’s movements (Hesse et al., 2012). Treatments were performed in three different modalities: (Bruni et al., 2018) passive mode (Bonan et al., 2004) active assistive mode and (Horak, 2006) active mode. Time to get in and out was 5 min. The net RASCT lasted 45 min/session. Patients were instructed to “help” the foot-plates gait-like movements during the training. The initial step-length, cadence and gait speed was individually set according to the spatiotemporal gait parameters measured by the GAITRite system (CIR Systems, Havertown, PA, United States) (minimum cadence: 30 step/min; gait speed range: 0.8–1.4 km/h; step height range: 12–18 cm; step cadence minimum: 14 step/min). The range of the body-weight support was between 50 and 0% to allow the patient to walk more symmetrically with higher velocities. It resulted in the facilitation of lower limb muscles and a more efficient gait (Hussein et al., 2008). Each training session consisted of robot-assisted gait training (15 min), robot-assisted stairs up (10 min) and down (10 min), passive lower limb joint mobilization and stretching exercises (10 min). The exercises (i.e., type of exercise, number of repetitions) and any adverse events that occurred during the study were recorded by the physiotherapist on the patient’s chart.

### The Sensory Integration Balance Training

The SIBT consisted of exercises aimed at improving the ability to integrate and reweight visual, proprioceptive and vestibular sensory input to maintain postural control. Each training session consisted of overground gait training (15 min), stairs up (10 min), and down (10 min), passive lower limb joint mobilization and stretching exercises (10 min). The net SIBT lasted 45 min/session. Exercises were repeated on a firm surface (floor) and compliant surfaces (i.e., mats of different section and resistance) (Smania et al., 2008; Gandolfi et al., 2014, 2015).

### Outcomes

At enrollment, clinical and demographic data were collected. A blinded examiner assessed primary and secondary outcomes before (T0), after treatment (T1) and 1 month after treatment (T2).

The primary outcome measure was the BBS. It is a validated 14-items measure for the assessment of static and dynamic balance in stroke patients. ICF domain: activity, maximum score: 54 (higher = better performance) (Cattaneo et al., 2006).

Secondary outcome measures were validated clinical scales to evaluate the training effects of electromechanical and robotic devices in post-stroke patients (Geroin et al., 2013). The Ten Meters Walking Test (10MWT) assessed the gait speed by measuring the time needed to walk 10 m and have been widely used in stroke patients. ICF domain: activity. A cut-off of 0.84 m/s has been reported to identify community ambulators (Bowden et al., 2008). The 6 min Walking Test (6MWT) assessed the distance walked over 6 min as a measure of endurance and aerobic capacity of the patient. It is commonly used in many neurologic conditions including stroke. ICF domain: activity. Normative data reported a score >400 m (Wevers et al., 2011). The Dynamic Gait Index (DGI) measured the patient’s ability to walk modifying balance according to external demands. Scores are based on a four-point scale, and tasks include steady-state walking, changing speed, turning head, overcoming obstacles and pivoting while walking and climbing stairs. ICF domain: activity, maximum score: 24 (higher = better performance) (Jonsdottir and Cattaneo, 2007). The TUG was used to measure of walking ability, balance, and fall risk in older adults. In the starting position patients sat on a chair and were asked to stand up, walk for 3 m at a self-selected speed, turn, walk back and sat on the same chair. The time between the starting position and the end of the task was recorded. ICF domain: activity. A minimal detectable change of 2.9 s was measured in patients with chronic stroke (Flansbjer et al., 2005). The Stair Climbing Test (SCT) assessed the ability to climb stair by measuring the time needed for the ascend and descend of stairs (nine steps). Step height was set at 20 cm. It has previously been used in the trial including subjects with cerebrovascular disease. ICF domains: activity (Harries et al., 2015).

The instrumental evaluation consisted of the stabilometric assessment using a force monoaxial platform (Stability System ST 310 Plus, Technobody). Patients were evaluated in the standing position without upper limb support. Feet position was standardized using a V-shaped frame, and patients were tested standing barefoot with arms alongside the body. According to the Sensory Organization Test protocol (Shumway-Cook and Horak, 1986), patients were assessed in six conditions, each lasting 30 s: (Bruni et al., 2018) stable surface with eyes open, (Bonan et al., 2004) eyes closed, and (Horak, 2006) dome condition; (Shumway-Cook and Woollacott, 2012) compliant surface with eyes open, (Smania et al., 2008) compliant surface with eyes closed, and (Peterka, 2002) compliant surface in dome condition (Shumway-Cook and Horak, 1986; Scoppa et al., 2013). In the dome condition, patients wore a visual-conflict dome positioned on patients’ head. An “*x*” sign was placed in the internal face of the dome aligned with the straight-ahead gaze of the patients. The dome moved in phase with patients’ head producing inaccurate visual orientation input (Shumway-Cook and Horak, 1986). This six-conditions test was used to examine the ability of the patients to maintain balance when sensory inputs were disrupted (Shumway-Cook and Horak, 1986). Data acquisition began after 10 s of patient’s familiarization with the task to limit non-stationary data, and the acquisition was stopped before patients were told to end the task (Da-Silva et al., 2016). The platform measures the position of the Center of Pressure (CoP) while subjects are standing on it with a sampling rate of 20 Hz. The coordinates of the Cop were used to calculate the length of the planar migration of the CoP over the platform (perimeter) [mm] and the sway area [mm^2^] in each condition. The sway area was computed as the area of the ellipse containing the 95% of CoP data points. Ellipse’s axes were calculated using principal component analysis (Oliveira et al., 1996). A platform integrated software computed Posturometric parameters.

### Sample Size

A sample size of 30 patients were necessary assuming α = 0.05 (probability of type 1 error) and a 95% power to detect a mean difference of 4.66 (DS 5.2) on the primary outcome measure (BBS) (Hiengkaew et al., 2012). Assuming a 10% drop-out rate, 32 patients were necessary to perform the study.

### Randomization

An automated randomization system^1^ was used to assign eligible patients to either the EG or the CG using. Group allocation was kept concealed. Only the principal investigator could access to the randomization list.

### Blinding

Primary and secondary outcomes were assessed by the same examiner blinded to the patient’s group allocation.

### Statistical Analysis

**FIGURE 2:**
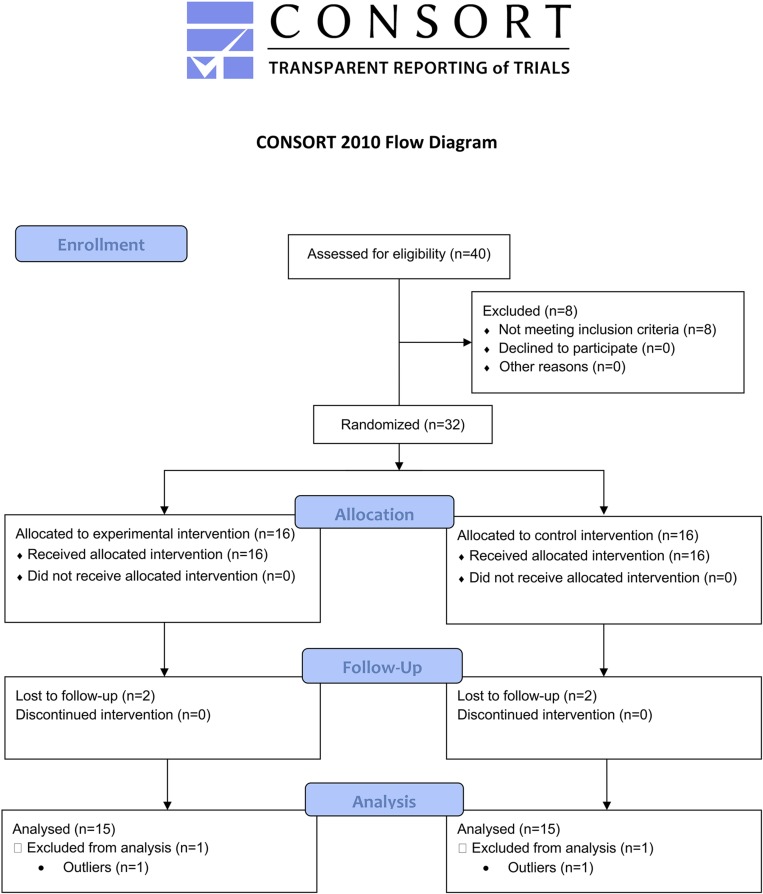
CONSORT flowchart.

An intention-to-treat analysis (Last Observation Carry Forward –LOCF-method) was used to handle missing data. Descriptive statistics included means and standard deviation. The *X*^2^ test was performed for categorical variables. Data distribution was checked to detect outliers (Figure 2). One patient in the EG and one patient in the CG differed significantly from the other observations (outliers) in all outcome measures and then they were excluded from the analysis. Data distribution was assessed with Shapiro–Wilk Test indicating a normal distribution. A two-way mixed ANOVA was used to analyze clinical outcome with Time as within-group independent variable, Group as between-group factor and the Time × Group factor to measure any interaction. Similarly, a two-way mixed ANOVA was used to evaluate the effects on the stabilometric assessment considering Group (×2) and Time (×3) factors and the six conditions for the analysis of stabilometric outcomes. Two-tailed Student’s *t*–test for unpaired data was used for between-group comparisons. The level of significance was set at *p* < 0.05. Bonferroni’s correction was applied for multiple comparisons. Statistical analysis was performed with SPSS 22.0 (IBM SPSS Statistics, Version 22.0, 2013, Armonk, NY, United States).

## Results

A total of 40 patients were assessed for eligibility: 8 were excluded because they did not meet inclusion criteria. Thirty-two patients were randomly assigned to either the EG (*n* = 16) or the CG (*n* = 16). Two patients in the EG and two in the CG were lost to follow-up (drop-out). Two patients were excluded from the analysis because they presented extreme values and, therefore, were considered outliers. No adverse events or safety concerns was reported during the conduction of the study.

Between-group analysis showed no significant differences in demographics and clinical data (Table 1) in primary and secondary outcome measures at baseline (T0).

**TABLE 1:** Demographic and clinical characteristics of patients.

	**Experimental Group (*n* = 16)**	**Control Group (*n* = 16)**	**Group comparison**
	
	**Mean (SD)**	**Means (SD)**	***p*-Value**
Age (years)	63.87 (11.44)	64.37 (10.56)	n.s.^a^
Sex M/F	10/6	13/3	n.s.^b^
BMI	26.49 (2.42)	26.19 (4.11)	n.s.^a^
Time from event (months)	54.81 (36.28)	53.06 (41.73)	n.s.^a^
Type of event I/E	13/3	13/3	n.s.^b^
Affection side L/R	6/10	4/12	n.s.^b^
European Stroke Scale (0–100)	72.12 (11.72)	72.56 (14.47)	n.s.^b^
Barthel Index (0–100)	90.93 (11.13)	90.62 (11.38)	n.s.^b^

*SD, standard deviation; M, male; F, female; BMI, body mass index; I, ischemic; E, hemorrhagic; L, left; R, right; ^*a*^, Mann-Whitney test; ^*b*^, chi square test.*

There was a non-significant main effect of group on primary and secondary outcomes (Table 2). A significant Time × Group interaction was measured on 6MWT (*p* = 0.013). Therefore, groups were analyzed separately. Overall significant changes over time were found in both groups (EG: *p* < 0.001; CG: *p* = 0.04). *Post hoc* analysis measured significant improvements in EG at T1 (*p* < 0.001) and T2 (*p* < 0.001). In CG significant changes were measured only at T2 (*p* = 0.008). An overall within-group improvement was found in 6MWT (*p* < 0.001), DGI (*p* < 0.001), 10 mWT (*p* < 0.001), TUG (*p* < 0.001) and SCT both in ascending (*p* < 0.001) and descending condition (*p* < 0.001).

**TABLE 2:** Descriptive and inferential statistics for clinical outcome measures.

		**Descriptive statistics**						**Repeated measure ANOVA**														
	**Group**	**T0**		**T1**		**T2**		**Time *P*-value**	**Time^∗^ Group *P-*value**	**Group *P-*value**	**Between-group comparison T0 IC 95%**				**Between-group comparison T1 IC 95%**				**Between-group comparison T2 IC 95%**			
		**Mean**	**SD**	**Mean**	**SD**	**Mean**	**SD**				***P-*value**	**Diff media**	**LB**	**UP**	***P-*value**	**Diff media**	**LB**	**UP**	***P-*value**	**Diff media**	**LB**	**UP**
BBS	EG	45.33	7.04	48.80	6.46	49.27	5.99	< 0.001^∗^	0.7	0.76	0.67	1.07	–4.04	6.18	0.81	0.53	–3.94	5.01	0.85	0.40	–3.79	4.59
	CG	44.27	6.62	48.27	5.47	48.87	5.17															
10 MWT	EG	12.76	5.53	11.08	5.09	10.72	4.82	< 0.001^∗^	0.71	0.77	0.65	–0.95	–5.26	3.35	0.95	–0.13	–4.22	3.96	0.72	–0.71	–4.66	3.24
	CG	13.72	5.97	11.21	5.82	11.43	5.71															
6 MWT	EG	203.4	80.1	237.9	95.3	234.3	90.1	< 0.001^∗^	0.013^∗^	0.49	0.75	10.73	–56.85	78.31	0.32	36.70	–37.42	110.82	0.49	24.47	–47.89	96.82
	CG	192.7	99.6	201.2	102.8	209.9	103.0															
DGI	EG	16.67	3.13	18.47	4.16	18.93	4.10	< 0.001^∗^	0.49	0.56	0.24	1.53	–1.09	4.16	0.76	0.47	–2.66	3.59	0.8	0.40	–2.76	3.56
	CG	15.13	3.85	18.00	4.21	18.53	4.34															
TUG	EG	22.81	8.65	19.17	8.14	18.59	8.44	< 0.001^∗^	0.41	0.94	0.73	–1.08	–7.50	5.33	0.89	0.37	–5.22	5.96	0.97	0.12	–5.91	6.15
	CG	23.89	8.50	18.80	6.74	18.47	7.67															
SCT Up	EG	13.10	4.53	10.72	4.01	10.72	3.87	< 0.001^∗^	0.51	0.83	0.99	–0.02	–3.50	3.46	0.85	–0.31	–3.73	3.11	0.68	–0.70	–4.17	2.76
	CG	13.12	4.78	11.03	5.08	11.42	5.29															
SCT Down	EG	13.50	4.55	10.98	4.37	10.73	4.34	< 0.001^∗^	0.37	0.43	0.52	–1.23	–5.10	2.63	0.53	–1.21	–5.07	2.65	0.33	–2.11	–6.48	2.25
	CG	14.74	5.72	12.19	5.84	12.85	7.01															

*SD, standard deviation; EG, experimental group; CG, control group; T0, pre-treatment; T1, post-treatment; T2, 1-month follow-up; BBS, berg balance scale; 10MWT, 10-meter walking test; 6MWT, 6-minute walking test; DGI, dynamic gait index; stocktickerTUG, timed-up-and-go TEST; CI: confidence interval; LB: lower bound; UB: upper bound; ^∗^, statistically significant; °, better performance.*

**FIGURE 3:**
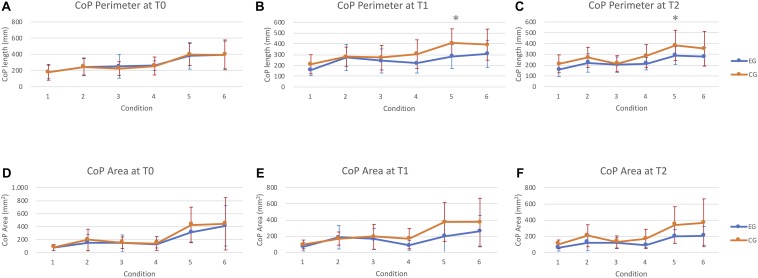
Instrumental assessment of postural control. **(A–C)** means ± standard deviation of CoP perimeter. Abscissa indicates the six conditions. Ordinate indicates the CoP perimeter (mm). **(D–F)** means ± standard deviation of sway area. Abscissa indicates the six conditions. Ordinate indicates the sway area (mm^2^). Asterisks indicates significant differences in between-group comparison.

The posturographic analysis showed a significant time × group interaction (*p* = 0.005). Therefore, groups were analyzed separately. Overall significant changes over time were measured in EG in CoP Perimeter in condition 2 (*p* = 0,015), 5 (*p* = 0,02), and 6 (*p* = 0,013). In contrast, no significant changes were measured in the CG. *Post hoc* within-group analysis showed a significant improvement at T1 in condition 5 (*p* = 0,024) and T2 in condition 6 (*p* = 0,008). Concerning the CoP Area, significant improvements were measured in EG in condition 4 (*p* = 0,014), 5 (*p* = 0,05), and 6 (*p* = 0,027). Significant differences were found at T2 in all conditions (4: *p* = 0,012; 5: *p* = 0,02; and 6: *p* = 0,012) but not at T1 (Figure 3 and Table 3).

**TABLE 3:** Descriptive and intra-group comparison of treatment effects.

								**Repeated measures ANOVA**	***Post Hoc* analysis - Mean within-group differences**							
**Outcome**	**Group**	**T0**		**T1**		**T2**			**IC 95% T0-T1**				**IC 95% T0–T2**			
		**Mean**	**SD**	**Mean**	**SD**	**Mean**	**SD**	**Time *p-*value**	***P-*value**	**Mean diff**	**LB**	**UP**	***P*-value**	**Mean diff**	**LB**	**UP**
**Perimeter CoP**																
Condition 1	EG	179.53	81.51	157.47	52.77	159.87	66.52	0.11	0.064	22.07	–1.50	45.63	0.111	19.67	–5.11	44.44
	CG	178.50	97.74	202.86	88.46	203.57	83.21	0.14	0.122	–24.36	–56.21	7.50	0.132	–25.07	–58.80	8.66
Condition 2	EG	245.47	110.74	272.80	122.33	215.67	84.93	0.015^∗^	0.042	–27.33	–53.51	–1.15	0.121	29.8	–8.94	68.54
	CG	245.93	101.21	277.36	88.57	267.00	94.14	0.29	0.182	–31.43	–79.63	16.77	0.399	–21.07	–73.24	31.09
Condition 3	EG	252.20	146.18	242.27	111.37	202.80	71.12	0.23	0.767	9.93	–60.63	80.50	0.155	49.40	–21.17	119.97
	CG	224.29	84.04	278.14	114.42	215.57	74.55	0.06	0.098	–53.86	–119.02	11.31	0.619	8.71	–28.26	45.69
Condition 4	EG	259.33	59.87	218.40	90.33	211.20	57.31	0.09	0.016^∗^	40.93	8.73	73.13	0.019^∗^	48.13	9.05	87.22
	CG	256.07	111.09	306.79	132.30	283.71	106.98	0.16	0.024^∗^	–50.71	–93.66	–7.77	0.242	–27.64	–76.41	21.12
Condition 5	EG	381.27	164.00	281.07	111.81	287.67	82.81	0.02^∗^	0.024^∗^	100.20	14.92	185.48	0.030	93.60	10.71	176.49
	CG	397.00	136.64	416.14	130.23	388.71	140.33	0.72	0.517	–19.14	–81.19	42.91	0.846	8.29	–82.13	98.70
Condition 6	EG	393.53	169.95	306.13	125.92	273.93	86.41	0.013^∗^	0.056	87.40	–2.67	177.47	0.008^∗^	119.60	37.04	202.16
	CG	393.00	184.78	404.71	144.07	362.14	160.45	0.56	0.812	–11.71	–115.94	92.51	0.427	30.86	–50.39	112.10
**Area CoP**																
Condition 1	EG	74.40	45.04	69.60	51.57	59.07	41.14	0.36	0.687	4.80	–20.26	29.86	0.091	15.33	–2.76	33.43
	CG	77.00	46.97	83.57	54.32	87.21	53.85	0.64	0.619	–6.57	–34.40	21.26	0.498	–10.21	–41.88	21.45
Condition 2	EG	152.07	127.69	186.87	144.97	121.07	103.00	0.08	0.187	–34.80	–88.64	19.04	0.289	31.00	–29.35	91.35
	CG	193.36	166.52	154.07	81.00	193.57	136.06	0.38	0.329	39.29	–44.38	122.96	0.995	–0.21	–68.65	68.22
Condition 3	EG	142.67	126.94	164.87	128.13	115.67	69.31	0.17	0.394	–22.20	–76.36	31.96	0.327	27.00	–30.02	84.02
	CG	150.64	88.02	185.50	153.51	119.21	74.79	0.13	0.281	–34.86	–101.87	32.15	0.069	31.43	–2.88	65.74
Condition 4	EG	129.53	60.58	86.53	60.69	85.33	24.52	0.014^∗^	0.026	43.00	5.81	80.19	0.012^∗^	44.20	11.02	77.38
	CG	139.00	107.42	153.07	125.49	152.29	119.39	0.78	0.529	–14.07	–61.07	32.93	0.585	–13.29	–64.49	37.92
Condition 5	EG	308.73	147.56	198.47	194.77	195.60	84.82	0.05^∗^	0.053	110.27	–1.57	222.11	0.021^∗^	113.13	19.03	207.24
	CG	424.36	275.20	361.00	240.39	322.36	225.86	0.35	0.399	63.36	–93.67	220.39	0.183	102.00	–54.52	258.52
Condition 6	EG	409.93	310.44	261.80	193.63	202.60	114.56	0.027^∗^	0.096	148.13	–29.90	326.17	0.012^∗^	207.33	51.76	362.91
	CG	441.93	403.59	378.36	295.79	371.64	295.61	0.56	0.554	63.57	–162.40	289.54	0.196	70.29	–41.22	181.79

*EG, experimental group; CG, control group; CoP, center of pression; T0, pre-treatment; T1, post-treatment; T2, 1-month follow-up; 1, open eyes - stable surface condition; 2, closed eyes - stable surface condition; 3, dome - stable surface condition; 4, open eyes - compliant surface condition; 5, closed eyes – compliant surface condition; 6, dome – compliant surface condition; ^∗^, statistically significant.*

## Discussion

The main finding of this RCT is twofold. Firstly, RASCT and SIBT produced comparable effects either to postural control or mobility in chronic post-stroke patients. Second, only the group that received the robot-assisted stair-climbing training reported significant improvements in the distance walked over 6 min and significant reduction of sway area and the CoP length on compliant surface in the eyes-closed and dome conditions.

Robot-assisted gait intervention offers the advantage of high-intensity and task-specific training that can be delivered, decreasing the physiotherapist physical burden (Morone et al., 2017; Sung et al., 2017). Over the last 20 years, the robot-assisted application in neurorehabilitation has inspired clinicians and researchers in further investigating the training effects on the multifaceted aspects involved in functional recovery after neurological disorders (Morone et al., 2017). A wide range of motor control dysfunctions might contribute to gait impairments in people with stroke. However, postural control disorders account for most of the gait-related disability such as problems with transferring, maintaining body position, mobility, and walking (Bruni et al., 2018). The development of evidence-based rehabilitation protocols is therefore of particular importance.

A pilot RCT study in 22 patients with Multiple Sclerosis (Expanded Disability Status Scale: 1.5–6.5) showed evidence that a robot-assisted gait training (Gait Trainer, Reha-stim, Berlin – Germany) might improve postural stability and the level of balance confidence perceived while performing Activities of Daily Lining (ADLs) as much as a sensory integration balance training (Gandolfi et al., 2014). For the first time, it has been suggested the various types of potential training effects of the end-effectors system in restoring gait function in people with a demyelinating disease. The hypothesis was that the robot-assisted approach would act as a form of “destabilization training” in the context of a “task-specific balance training” by the end-effector system. Destabilization training includes tasks that induced unexpected external or internal destabilizations of the center-of-body mass (CoP), while patients are asked to keep the standing posture. Our findings cannot be fully discussed with those by Gandolfi et al., 2014 due to differences about patients and the type of the robot-assisted device. However, our results confirm these literature findings in patients with chronic stroke.

The two interventions showed comparable effects on static and dynamic activities of varying difficulty, on the ability to modify balance while walking in the presence of external demands and on mobility as assessed by the clinical scales. Note that, neither the experimental nor the control group achieved the minimum clinical significance change of five points post-treatment in the primary outcome measure (Donoghue et al., 2009). The BBS is psychometrically robust (Tyson and Connell, 2009a) and very sensitive to exercise intervention in neurological population (Pedroso et al., 2012). However, we did not measure clinically significant changes. Therefore, we could not exclude accustoming effects during the training or ceiling effects. Similarly, both interventions improved walking speed over a short duration, as evaluated by the 10-meter walking test. In the framework proposed by Horak (2006) both pieces of training might have exerted their effects improving movement and sensory strategies (sensory integration and reweighting), orientation in space and control of dynamic (Horak, 2006) acting as task-specific balance training (Gandolfi et al., 2014). The robot-assisted training in addition may have improved proprioception, and the integration of proprioceptive and vestibular sensory input, in standing on compliant surface (condition n. 4-5-6) (Gandolfi et al., 2014). A possible explanation is that the robotic approach might have reinforced the neural circuits that contribute to face postural adjustments. The G-EO system is an end-effector system (Hesse et al., 2012). In this context, a reduced number of constraints interact with the patients allowing freedom, especially for ankle and hip movements, on a mobile base of support. The gait-like footplates movement might shape ankle strategies required to maintain balance for small amounts of sway when standing on a firm surface (Horak and Kuo, 2000). The lack of constraints, especially for the pelvic movement, might account for hip strategies improvements, in which the body exerts torque at the hips to quickly move the body CoM, is used to stand on narrow or compliant surfaces that do not allow adequate ankle torque (Horak and Kuo, 2000). The fact that the physiotherapist set the step and gait parameters (i.e., step length and pace) according to the patient’s improvements emphasized the progression of the task demand. Moreover, the passive training mode might have been improved Compensatory Postural Adjustments (CPAs – Feedback mechanisms) by providing high-intensity and repetitive external destabilization. Note that, the stair climbing protocol might have further strengthened these effects enhancing the amplitude of the external perturbation on different planes (climbing up and down). Stair climbing up and down can be seen as a repeated sequence of balance challenges that rarely can be applied in patients with stroke because of the danger of the task. Negotiating stairs is a typical community ambulation requirement and the final goal of the rehabilitation plan, as the hallmark of complete recovery of mobility in the environment. Challenges of stair climbing, and level walking in the same rehabilitation session under different (active, passive and robot-assisted) training modalities allow to train specifically reactive, anticipatory and voluntary movement strategies (Emken and Reinkensmeyer, 2005; Horak, 2006; Lam et al., 2006; Gandolfi et al., 2014). Stair climbing is demanding from a neuromuscular (Nadeau et al., 2003) and metabolically point of view (Modai et al., 2015). Interestingly, only the robot-assisted group showed a clinically significant improvement in the distance walked over 6 min reaching the MCID value after treatment (34.53 m), as a proof of aerobic capacity/endurance improvements. According to the literature, stair climbing training can improve post-stroke aerobic capacity (Nadeau et al., 2003; Modai et al., 2015). In the context of conventional rehabilitation training, it is not possible to train postural reaction passively as well as intensive and repetitive stair climbing training.

An important issue that required discussion was the chronic stages of the illness. Literature has highlighted that in the chronic stage of stroke, the brain is relatively likely to support endogenous recovery. However, modifications in brain structures and function are still possible after specific interventions (Cramer, 2018). For patients with severe lower limb impairment, robotic training produces better outcomes than conventional training (Lo et al., 2017). Thus, results might be affected by the fact that enrollees had mild motor deficits, as measured at the enrollment. To date, no normative data on time to climb up and down stairs are available in the literature.

The strengths of the present study are the low drop-out rate confirming the feasibility of training in patients with chronic stroke. The comprehensive assessment of postural control using validated and psychometrically robust measures, and instrumental assessment are further strengths of this study (Tyson and Connell, 2009a, b). However, the use of clinical balance outcome measures specific to explore the underlying sensorimotor mechanisms contributing to the balance training effects (i.e., Mini Best Test) should have explored more specifically the training effects (Mancini and Horak, 2010). The study limitations are the lack of a real control group without any intervention, the use of functional balance assessment (i.e., BBS) instead of a system approach (i.e., Mini Best Test) and the lack of patient with more severe neurological impairment. Future studies should evaluate the training effects on participation and quality of life.

To conclude, RASCT is a feasible and valid approach to improve postural control and mobility in patients with chronic stroke. Robotics held promise and ensured to enrich rehabilitation when combined with sensory integration balance training. The advantages of combined training might be beneficial to overcome their limits. The present study is an effort to provide a reference for robot-assisted balance training protocols. Issues such as optimal dosage according to the degree of neurological disability need still to be addressed.

## Data Availability Statement

The datasets for this manuscript are not publicly available because CRRNC property. Requests to access the datasets should be directed to MG, marialuisa.gandolfi@univr.it.

## Ethics Statement

The study was conducted according to the tenets of the Declaration of Helsinki, the guidelines for Good Clinical Practice, and the Consolidated Standards of Reporting Trials (CONSORT), approved by the local Ethics Committee (1442CESC). Clinical trial registration NCT03566901.

## Author Contributions

MG and ED have made substantial contributions to conception and design. EB and AP participated in the enrollment phase. NM and CG carried out the clinical assessment and instrumental assessments. MB and ED carried out the rehabilitation treatments. MG and NV participated in the statistical analysis and drafted the manuscript. MZ revised the statistical analysis. NS and AW participated in the manuscript revision process and gave the final approval of the version.

## Conflict of Interest

The authors declare that the research was conducted in the absence of any commercial or financial relationships that could be construed as a potential conflict of interest. The handling editor declared a past collaboration with one of the authors, NS.

## Footnotes

1 http://www.randomization.com
